# *Phyllopertha horticola* (Coleoptera: Scarabaeidae) larvae in eastern Austrian mountainous grasslands and the associated damage risk related to soil, topography and management

**DOI:** 10.1186/s40064-015-0918-6

**Published:** 2015-03-24

**Authors:** Patrick Hann, Claus Trska, Katharina F Wechselberger, Josef Eitzinger, Bernhard Kromp

**Affiliations:** Bio Forschung Austria, Esslinger Hauptstrasse 132-134, Vienna, 1220 Austria; MELES GmbH, Mörikestraße 20, St. Pölten, 3100 Austria; Department of Water, Atmosphere and Environment, University of Natural Resources and Life Sciences, Vienna, 1180 Austria

**Keywords:** *Phyllopertha horticola*, *Hoplia philanthus*, *Melolontha melolontha*, Humus, White grubs, Cutting frequency

## Abstract

The soil-dwelling larvae of several Scarabaeidae species (white grubs), like the cockchafer (*Melolontha melolontha*) and the garden chafer (*Phyllopertha horticola*), are serious pests in European cultivated grassland, reducing grass yield and destroying the turf by root-feeding. Nevertheless, the factors responsible for the development of large grub populations and the associated damage risk are poorly understood. The objectives of the study were to survey grub densities in grassland sites with different damage histories and find correlations with environmental and management variables. Data on grub densities were collected at 10 farms in the eastern Austrian Alps in September and October 2011. At each farm, one recently damaged site (high risk) and one site at which grub damage had never been observed by the farmers (undamaged site = low risk; each site: 500 m^2^) were sampled. All sites were dominated by *P. horticola* (99% of 1,422 collected individuals; maximum density 303 grubs/m^2^), which indicates that grub damage there is mainly caused by that species. Recently damaged sites tended to higher grub densities than undamaged sites. However, 3 out of 10 undamaged sites harbored high grub populations as well. Humus content together with the depth of the A-horizon significantly explained 38% of *P. horticola* grub density variance, with highest densities in deeper humus-rich soils. The risk of grub damage was positively connected to the humus content and negatively related to the cutting frequency. For the investigated mountainous grassland sites, these results suggest an important role of humus for the development of high grub densities and an effect of management intensity on grub damage.

## Introduction

White grubs, the soil-dwelling larvae of Scarabeidae species, cause severe damage to European cultivated grassland (Keller and Zimmermann 2005, Jackson and Klein 2006). Heavy grub feeding to the grass roots reduces the grass yield and can endanger farmers by causing their farm machines to slip down slopes on the detached sward. Based on a nationwide survey conducted in 2000, Strasser (2004a) forecasted increasing damage caused by Scarabaeidae larvae in several Austrian regions. In a survey, performed by interrogating plant protection consultants of 74 Agricultural County Chambers all over Austria, a cumulated area of 14,800 ha of white grub damage were recorded for the investigation period from 2000 to 2006 (Grünbacher et al. 2007, Hann et al. 2008).

According to the literature, the Scarabeidae (Coleoptera) species, mainly responsible for grub damage in Austrian alpine grasslands, are the cockchafers (*Melolontha melolontha* and *M. hippocastani*), the garden chafer (*Phyllopertha horticola*) and, to a lesser degree, the June beetle (*Amphimallon solstitiale*; Pötsch et al. 1997, Traugott 2003, Strasser 2004a, Keller and Zimmermann 2005). Keller (2004) reported *A. solstitiale* to cause damage mainly in sports turfs, parks and gardens. Also the survey results of Strasser (2004a) indicated that particularly *M. melolontha* and *P. horticola* cause increasing damage in cultivated grasslands all over Austria, while *A. solstitiale* is of lesser significance. Since the adults of *A. solstitiale* might be often mistaken for *M. melolontha* beetles, the contribution of this species to damage in managed pastures could as well be underestimated (Traugott and Juen 2008).

According to Pötsch et al. (1997), the areas damaged by the cockchafers are rather found in the valleys and plains of 300 to 600 m above sea level, while the areas damaged by the garden chafer extend from the valleys up into higher mountainous regions. This observation corresponds to Scheerpelz (1950), who stated that *M. melolontha* mainly occurs in areas with annual mean temperatures of at least 7°C. *Melolontha melolontha* (adult: 20–30 mm) has a three or four year life cycle in Austria, while the smaller *P. horticola* (adult: 8.5–12 mm) completes its larval development within one year (Raw 1951, Freude et al. 1969, Pötsch et al. 1997). Due to its shorter life cycle, *P. horticola* can build up high population densities more quickly in response to favourable environmental conditions. Faber (1961) discovered a transition from a four-year to a three-year development cycle of *M. melolontha*, particularly in some Alpine valleys. This trend is probably connected to increasing mean temperatures (Keller 1993, Scheifinger et al. 2007). However, recent systematic grub density surveys in damaged Austrian grasslands are not reported in the literature.

Since the larvae of the problematic Scarabaeidae species are soil dwelling, their population densities and the associated damage risk are probably depending on site characteristics that influence soil temperature, soil moisture and resilience of the sward, like grain size, humus content or management impact on vegetation coverage. Although this hypothesis is supported by observations reported in the literature, the environmental conditions that are determining grub populations and the damage risk in grassland sites are poorly understood.

Zweigelt 1928 (quoted by Scheerpelz 1950) described climatic and soil conditions as main factors responsible for high densities of *Melolontha ssp.* grubs. Similarly, population fluctuations of *P. horticola* can be attributed to climatic factors (Milne 1964 and 1984). Laznik et al. (2012) stated that grubs are most common in sandy or sandy loam soils, but can also occur in clay soils. According to Scheerpelz (1950), Pötsch *et al.* (1997) and Albert and Fröschle (2010), for oviposition, *M. melolontha* prefers dry, loose soils and sunny meadows, particularly with a slightly gappy sward and a high temperature radiation which might direct the female beetles to suitable locations. Furthermore, for the optimal development of the larvae warm, dry, moderately permeable, deep and eutrophic soils are needed (Scheerpelz 1950). In contrast, Faber (1951a) stated that the locations with not too high, but closed vegetation are selected for oviposition. Similarly to *M. melolontha*, *P. horticola* prefers dry and sunny habitats with sandy soils and slightly gappy swards, while dense swards with high vegetation inhibits the egg deposition of the females (Milne 1964, Bocksch 2003). In British grassland sites, Raw (1951) observed a preference of *P. horticola* adults for dense, unscythed vegetation, but reports that more grubs of the species could be found in dry friable than in moist soils. Apart from a possible effect on egg deposition, a dense, well supplied sward reduces soil warming and might endure grub feeding to the roots much better than weak grass plants. Accordingly, management measures that support a dense sward, like a balanced cutting, grazing and manuring regime, are considered to reduce the risk of grub damage (Pötsch et al. 1997).

The control of Scarabaeidae larvae by means of chemical pesticides is quite difficult because of their cryptic habitat. Apart from problems with application into soil, there is growing concern about safety and environmental contamination linked to the usage of chemical pesticides (Jackson and Klein 2006). Approaches to organic grub control are the application of nematode and entomopathogenic fungi products, like Melocont® (*Beauveria brongniartii*) against *M. melolontha* grubs and GranMet-P® (*Metarhizium anisopliae*) against *P. horticola*. After application, the fungus-epizootic needs time to spread in the soil (Strasser 2004b, Keller and Zimmermann 2005, Jackson and Klein 2006). A grub damage risk forecasting system based on site characteristics and climate would enable the farmers to take measures in time. However, the development of such a system requires better knowledge of the relationships between site, climate and management factors, grub densities and damage.

The main objective of our study was to investigate the factors that determine the risk of grub damage in mountainous agricultural grasslands. Therefore the relevant tasks were to i) survey grub densities in grassland sites to reveal the dominant species and find correlations with environmental and management variables and ii) to compare damaged and undamaged grassland sites to reveal site and management characteristics that increase the risk of grub damage.

## Materials and methods

### Sampling sites

**Figure 1:**
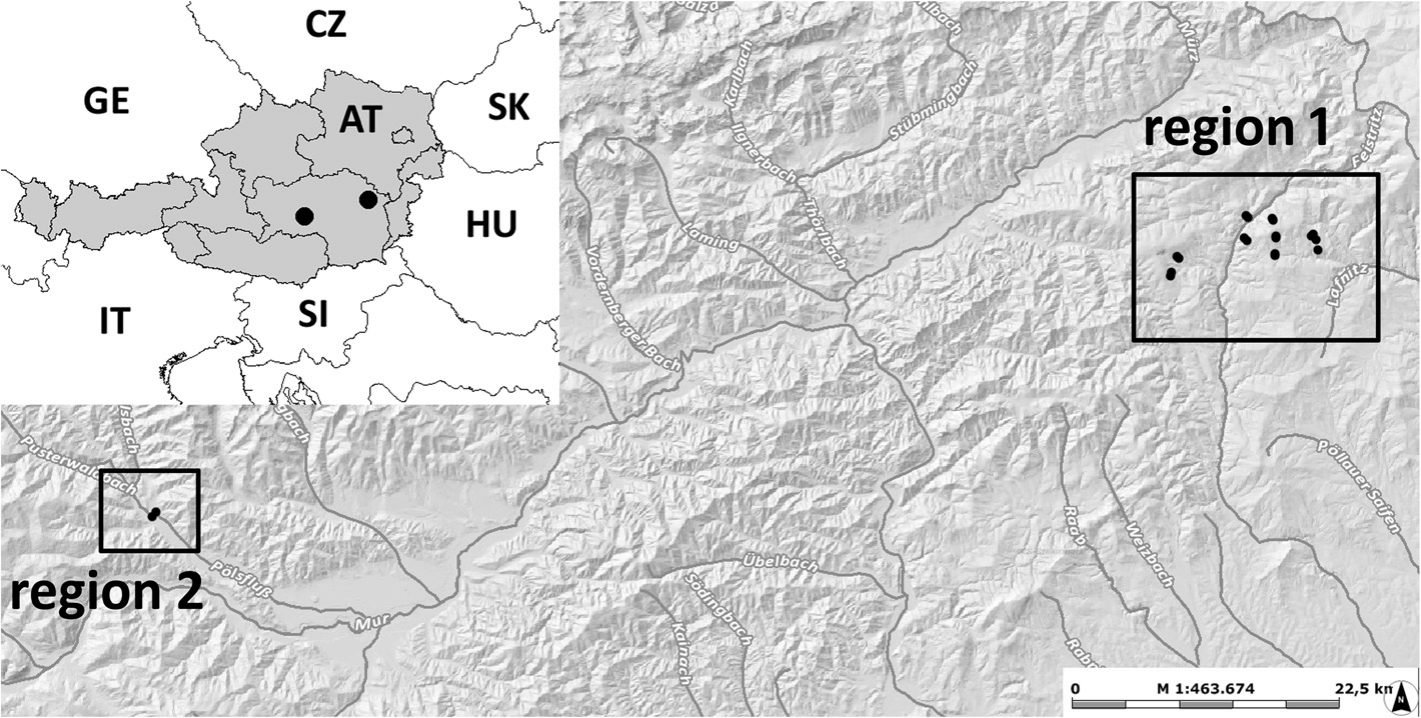
**Situation of the grub sampling sites in eastern Austria.** The sites are indicated by small black dots: region 1 = south-eastern alpine foothills, region 2 = central-eastern alps. The small map in the upper left corner shows the situation of the sampling regions within Austria/Europe (large black dots); AT = Austria, CZ = Czech Republic, GE = Germany, HU = Hungary, IT = Italy, SI = Slovenia, SK = Slovakia (www.gis.steiermark.at).

We examined two sampling regions (Figure 1), which were affected by grub damage, over the last decade. Region 1 was situated in north-eastern Styria, covering the upper Feistritz valley (district Weiz) and the upper Lafnitz valley (district Hartberg). This region had a north–south extent of about 8 km and an east–west extent of about 14 km and contained 18 of the 20 sampling sites. The second sampling region (region 2) was situated about 90 km west of region 1 in central-western Styria (upper Mur valley, district Murtal). It contained only two sites with a distance of about 500 m.

Both regions were mountainous and dominated by agricultural grassland. The altitudes of the sampling sites varied between 839 and 1,104 m above sea level in region 1 and 898 and 906 m in region 2. The climatic characteristics of the regions are shown in Table 1. The sites in both regions were characterized by sandy, lime-free Leptosols or Cambisols over siliceous, gravelly material (eBOD, 2013).

**Table 1:** **Climatic characteristics (1971 – 2000) of the two sampling regions, measured at representative weather stations (ZAMG** 2014 **)**

	**Region 1**	**Region 2**
Weather station	Fischbach	Oberzeiring
Coordinates of the station	47°27'N, 15°53'E	47°15'N, 14°29'E
Altitude (m above sea level)	1,050	930
Situation of the station	North-eastern slope	Valley, southern slope
Mean temperature (°C)	13.12	13.04
Mean precipitation sum per month (mm)	583.0	525.2

The measured data were averaged across the adult flight and main larval feeding period (May – September) of *Phyllopertha horticola* (Scarabaeidae).

**Figure 2:**
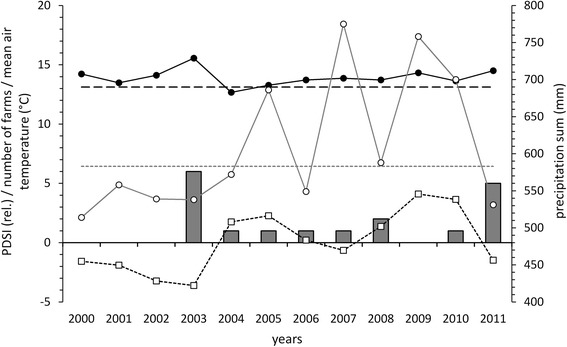
**Annual weather conditions and the occurrence of grub damage since 2000 in region 1.** Mean air temperature, mean precipitation sum and mean drought index (PDSI) during adult flight and main larval feeding period (May – September) of *Phyllopertha horticola* (Scarabaeidae), measured at the ZAMG weather station in Fischbach (47°27'N, 15°53'E, 1050 m above sea level; ZAMG 2014), as well as the number of investigated farms in sampling region 1 (near Fischbach, 9 farms in total) that reported grub damages in the respective year (grey bars); solid black line with black circles = annual mean air temperatures, solid grey line with white circles = annual precipitation sums, dashed black line = mean air temperature from 1971 to 2000 (May – September), dotted grey line = mean precipitation sum from 1971 to 2000 (May – September), dotted black line with white squares = annual Palmer Drought Severity Index (PDSI), ranging from +5 = extreme wetness to −5 = extreme drought.

The individual sampling sites were determined in two steps. First, we selected 10 farms which had recently reported grub damage (without knowing the grub species) and, in the case of region 1, were evenly distributed over the sampling region (Figure 1, Figure 2 and Table 2). At each farm, we asked for two sites: a grassland site which had recently been damaged by grubs (= damaged sites) and a second site at which grub damage had never been observed by the farmers (= undamaged sites; mean distance between the two sites: 315 m; range: 180 – 836 m). We postulated that: 1) The observations of farmers regarding grub damage were highly reliable, because grub damage meant difficulties in management, economic loss and even danger, since machines can slide on a detached sward. 2) The risk of grub damage is higher at sites where grub damage had recently occurred (damaged sites = high risk) than at sites at which damage had never been observed by the farmers (undamaged sites = low risk). Our approach assured that the sampling sites met two important requirements for our study. Each site had a grub infestation potential, since grub damage had been reported recently, at least in its vicinity, and the sites covered two grub damage risk levels (low risk and high risk). This was important for investigating relationships between grub damage risk, environmental and management variables.

**Table 2:** **Localisation of the sampling sites and collected data on grub densities and damage; years = years from 2000 to 2010 in which damage was observed by the farmers**

					**Coordinates (UTM 33 N, WGS84)**		**Grub damage**				**Grubs per species/m** ^**2**^		
**Sample**	**Region**	**Farm**	**Field**	**Sampling date**	**Easting**	**Northing**	**Before 2011**	**Years**	**In 2011**	**grubs/m** ^**2**^	***P. horticola***	***H. philantus***	***Melolontha sp.***
11	1	1	1	2011-09-06	548047	5254667	yes	2003, 2010	yes	113.2	113.2	0.0	0.0
12	1	1	2	2011-09-06	548141	5254924	no	/	no	71.9	71.9	0.0	0.0
21	1	2	1	2011-09-06	548602	5256315	yes	2003, 2006	yes	205.2	204.2	0.0	1.0
22	1	2	2	2011-09-06	548678	5256179	no	/	no	4.2	4.2	0.0	0.0
31	1	3	1	2011-09-07	560089	5258184	no	/	no	37.5	37.5	0.0	0.0
32	1	3	2	2011-09-07	559905	5258072	yes	2003	yes	303.1	303.1	0.0	0.0
41	1	4	1	2011-09-14	556813	5256383	yes	2003	no	33.3	33.3	0.0	0.0
42	1	4	2	2011-09-14	556829	5256594	no	/	no	1.0	1.0	0.0	0.0
51	1	5	1	2011-09-23	556937	5257877	yes	2007, 2008	no	60.4	60.4	0.0	0.0
52	1	5	2	2011-09-23	556903	5258062	no	/	no	50.0	50.0	0.0	0.0
61	1	6	1	2011-09-22	560243	5257713	no	/	no	1.0	1.0	0.0	0.0
62	1	6	2	2011-09-22	560385	5256908	yes	2003, 2004	yes	94.8	94.8	0.0	0.0
71	1	7	1	2011-09-28	554443	5257666	no	/	no	3.1	3.1	0.0	0.0
72	1	7	2	2011-09-28	554287	5257875	yes	2008	no	71.9	71.9	0.0	0.0
81	1	8	1	2011-09-27	556555	5259566	yes	2003	no	26.0	26.0	0.0	0.0
82	1	8	2	2011-09-27	556601	5259399	no	/	no	13.5	13.5	0.0	0.0
91	1	9	1	2011-10-03	554569	5259652	yes	2005	yes	97.9	97.9	0.0	0.0
92	1	9	2	2011-10-03	554398	5259694	no	/	no	95.8	78.1	17.7	0.0
101	2	10	1	2011-10-04	462434	5234317	yes	n/a	yes	167.7	167.7	0.0	0.0
102	2	10	2	2011-10-04	462773	5234718	no	/	no	30.2	30.2	0.0	0.0

**Figure 3:**
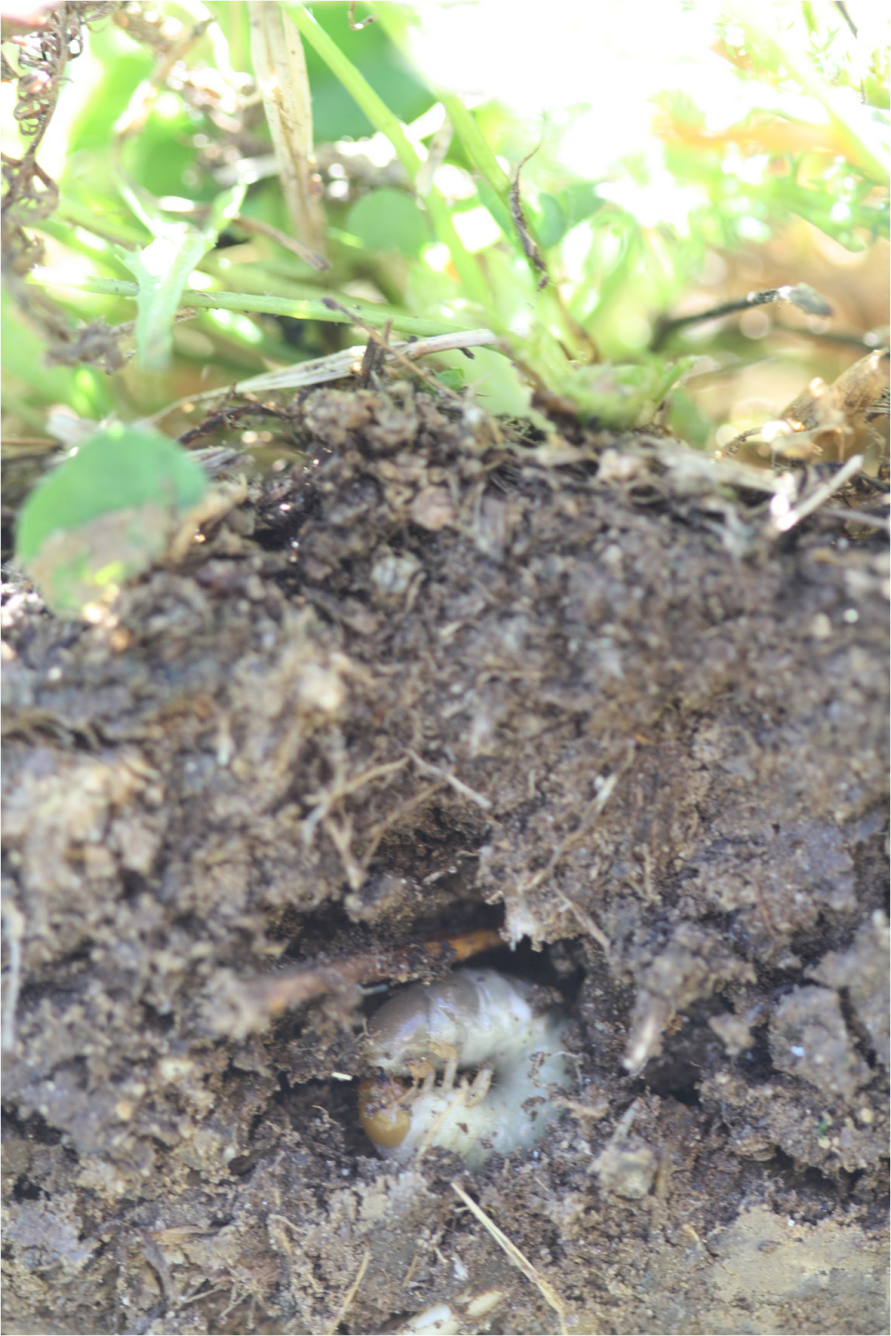
***Phyllopertha horticola*** **(Scarabaeidae) larva in situ.**

Each sampling site covered an area of 500 m^2^. For measuring grub density, 24 soil samples were taken evenly distributed across this area. These subsamples had an extent of 20 × 20 cm and reached about 10 cm into the topsoil (A-horizon), since the common grub species in Middle European grasslands (*M. melolontha, P. horticola, A. solstitiale, Hoplia philanthus*) are usually located not deeper than 10 cm during summer and autumn feeding (Raw 1951, Milne 1956, Hasler 1986, Keller 2004, Benker and Leuprecht 2005). The subsamples were cut out with a spade and searched on a tablet for grubs. All grubs were counted: all undamaged grubs were collected for determining the species (Klausnitzer 1996). The samplings were conducted from September 1^st^ until October 4^th^ 2011. At this time, the grubs were feeding on the grass roots a few millimeters to centimeters beneath the soil surface. Thus, the grubs could easily be found (Figure 3).

In addition to estimating the grub densities, we also recorded current grub damage in 2011 (yes/no). In order to obtain material for soil chemical analyses, we took five evenly distributed soil samples (0 – 10 cm) per site. The soils were analyzed by Bio Forschung Austria for the contents of humus (%), N_min_ (kg/ha), and dissolved organic carbon (DOC, kg/ha) as well as the pH-H_2_O. Further soil characteristics were obtained from the eBOD database (http://gis.lebensministerium.at/eBOD): maximum depth of the A-horizon (cm), humus content eBOD (%; derived by the IKT Petzenkirchen) and water supply (= estimated values: 1 = very low to 7 = very high). Topographical data were derived from the Styrian GIS system (www.gis.steiermark.at): altitude (m above sea level), slope (degrees) and aspect (degrees). The soil texture (percentage of sand, silt and clay) of the soil samples was analysed by the IKT-Petzenkirchen (A-3252 Petzenkirchen). Furthermore, the management regime and the detailed damage history of each site was recorded by interrogating the respective farmers: the usual numbers of cuts and grazings and the usual amount of applied manure (0 = none, 1 = low, 2 = medium, 3 = high) per year.

### Statistics

The data were statistically analyzed using SPSS 11.5.1 (SPSS Inc., Chicago, IL, USA). An error probability of p ≤ 0.05 was considered as statistically significant, 0.05 < p ≤ 0.1 was considered as trend and 0.1 < p ≤ 0.15 as weak trend. For each sampling site, the grub numbers per subsample and species were averaged. To assess the accuracy of the estimated mean grub densities per site, the confidence intervals (95%, p = 0.05) were calculated.

Differences between damaged and undamaged sites considered grub density (= all species) and *P. horticola* density, environmental and management variables were analyzed by t-tests or by Mann–Whitney-U-tests, in case of nonparametric data.

Relationships between *P. horticola* density and management, as well as environmental variables, were modeled and tested by a stepwise multiple linear regression analysis. Additionally, Pearson correlation analyses and, in case of non-parametric data, Spearman rank correlation analyses were conducted. The probability of grub damage, i.e. the probability that a site belonged to the category that had been recently damaged by grubs (= high risk) as reported by the farmers, was modeled by a stepwise multiple logistic regression analysis in dependence of management and environment.

To investigate the influence of grub feeding activity on the humus content of the soil, the relationship between the humus contents in the soil samples and the corresponding eBOD humus contents, which had been derived from a soil map and were therefore unaffected by grub densities, was modeled by a linear regression analysis. In a second step, the effect of grub feeding on this relationship was evaluated by testing the explanatory contribution of the parameter “grub density per m^2^” (all species), when added to the linear regression model.

Normal distribution was tested by the Kolmogorov–Smirnov-Test. If necessary, data were transformed by natural logarithm (ln(x)), square root (x^2^), division (1/(x*-1)) or, in case of percentages, by arcussinus (arsin√(x/100)), to meet statistical requirements.

In order to depict annual drought conditions in region 1 (ZAMG station Fischbach, see Table 1), we calculated the monthly Palmer Drought Severity Index (PDSI), using historical climate data from 1990 to 2012. The PDSI ranges from extreme wetness (index value: +5) to extreme drought (index value: −5; Palmer 1965, Dai et al. 2004). For each year, the index was averaged over the adult flight and main larval feeding period of *P. horticola* from May to September (Milne 1959).

## Results

In 2003 and in the sampling year 2011, high proportions of the investigated farms in the region around Fischbach (region 1) were affected by grub damage (2003: 67% of all investigated farms, 9 farms in total; 2011: 56%, Figure 2). Both years were characterized by comparatively high mean temperatures and low precipitation sums during the adult flight and main larval feeding period of *P. horticola* (May – September). Accordingly, in both years the Palmer Drought Severity Index value was lower than zero, indicating drought conditions. The years before both *P. horticola* outbreaks showed high mean temperatures as well, whereas their precipitation sums were diverging with low values in 2001 and 2002 but high values in 2009 and 2010. Mean temperatures in the region were almost continuously higher than the long term average from 1971 to 2000, with only one exception in 2004.

### Differences between damaged and undamaged sites concerning grub density, environmental variables and management

**Figure 4:**
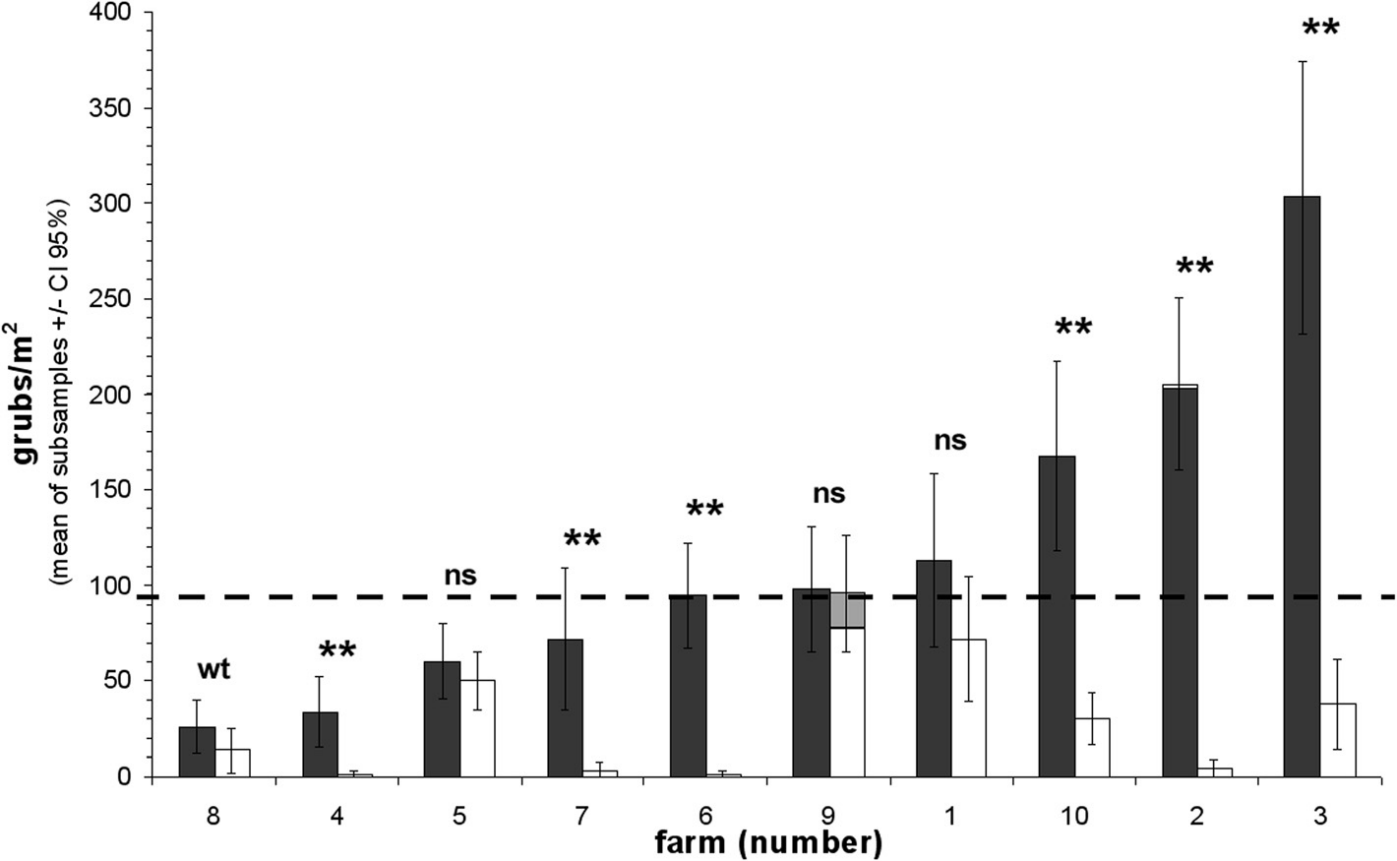
**Total mean grub densities per sampling site (+/− confidence interval, p = 0.05).** dark bars = sites had recently been affected by grub damage (damaged sites), white bars = grub damage had never been observed by the farmers (undamaged sites); species composition was dominated by *Phyllopertha horticola* except: light grey section in the white farm 9 bar = mean number of *Hoplia philanthus*, white section at the top of the dark farm 2 bar = mean number of *Melolontha sp.*; Statistical symbols according to t-tests and Mann–Whitney-U tests, in case of non-parametric data: **p ≤ 0.01, wt = weak trend = 0.1 < p ≤ 0.15, ns = non-significant; The dashed line marks the grub density level, above which grub damages were visible in 2011.

As shown in Figure 4, a wide range of grub densities was sampled, ranging from 1 to 303 individuals/m^2^. Since the confidence intervals indicated a high accuracy of the mean grub densities per site, meaningful site comparisons could be expected. The sites, that had recently shown visible grub damage (dark grey) mainly tended to higher grub densities than the respective sites without noticeable damage (white). A *t*-test calculated across all farms confirmed the difference between the two site categories to be highly significant (p = 0.002). However, at some individual farms (1, 5, 8, 9) the differences were not significant. The collected grubs were largely determined as the garden chafer (*P. horticola* = 99% of 1,422 collected individuals in total). The grub density threshold, above which grub damage in the sward was visible in late summer and early autumn 2011, lay at about 94 grubs/m^2^. The only site with a higher mean grub density that showed no damage was the “undamaged” site at farm 9 (= site 92, white bar). This was the only site, at which a significant part of the grubs was not determined as *P. horticola* but as *Hoplia philantus*. Visible grub damage was recorded in 2011 only at sites that had already been damaged by grubs before our study (= damaged sites). Comparing the two site categories based on management and environmental variables showed that the usual number of cuts was significantly higher at the undamaged than at the damaged sites (Table 3). Cutting frequency was significantly positively correlated with the usual amount of applied manure (r = 0.51, p = 0.023; Table 4) and significantly negatively correlated with the usual number of grazings (p = − 0.56, p = 0.010). However, neither of these variables (amount of applied manure, number of grazings) showed significant differences between damaged and undamaged sites. On the contrary, the DOC contents were significantly higher at the damaged than at the undamaged sites. Humus content tended to have a similar result with a higher mean value for the damaged sites. No other measured variable showed differences. DOC and humus contents were weakly, but significantly positively correlated (r = 0.49, p = 0.030).

**Table 3:** **Comparison of the “undamaged” and the “damaged” sites regarding grub density, environment and management; site categories: “undamaged site” = grub damage had never been observed by the farmers (low risk) and “damaged sites” = grub damage had recently occurred (high risk)**

**Site categories**		**Undamaged sites (= low risk)**				**Damaged sites (= high risk)**		
	**Minimum**	**Maximum**	**Mean/median**	**+/− SEM**	**Minimum**	**Maximum**	**Mean/median**	**+/− SEM**
Grubs (all species) per m^2^**	1.04	95.83	30.83	10.47	26.04	303.13	117.63	27.16
Grub damages 2011 (ord)	0	0	0	/	0	1	1	/
Humus (%) t	4.57	11.95	8.13	0.72	5.89	12.53	9.93	0.67
N_min_ (kg/ha)	5.00	36.00	11.90	2.81	7.00	24.00	13.20	1.50
DOC (kg/ha)*	48.00	238.00	102.80	17.07	86.00	273.00	154.20	21.96
pH-H_2_O	5.16	6.13	5.53	0.10	5.15	5.75	5.49	0.06
Clay (%)	13.08	21.10	15.94	0.74	11.90	22.83	16.44	1.03
Silt (%)	29.60	42.58	34.39	1.27	27.95	38.31	33.15	1.00
sand (%)	41.06	57.26	49.65	1.81	38.86	56.78	50.39	1.75
A-horizon max. (cm) eBOD	20.00	30.00	24	1.25	15.00	30.00	24	2.21
Humus db (%) eBOD	2.20	4.90	3.34	0.31	2.20	5.10	3.79	0.35
Water supply (ord) eBOD	3.00	7.00	5	/	3.00	5.00	5	/
Altitude above sealevel (m)	838.86	1103.99	983.68	25.68	850.93	1094.65	966.47	24.67
Slope (degrees)	4.92	22.24	13.03	1.71	3.07	24.13	14.53	1.85
Aspect (degrees)	3.19	169.62	66.55	15.21	2.32	147.28	74.86	17.01
Usual number of cuts/year*	1.00	4.00	2.10	0.23	0.00	2.00	1.40	0.22
Usual number of grazings/year	0.00	2.00	1.00	0.15	1.00	4.00	1.60	0.40
Usual manure amount/year (ord)	1.00	3.00	1.5	/	0.00	3.00	1	/

For parametric data the means (+/− SEM = standard error of the mean), in case of ordinal parameters (ord) the medians were calculated. Significant differences between the two site categories are indicated by: ** = p ≤ 0.01, * = 0.05 ≥ p > 0.01, t = 0.1 ≥ p > 0.05 trend. The p-values were calculated by t-tests or Mann Whitney U-tests in case of ordinal variables. Parameters: The soil parameters were analysed from soil samples (0 – 10 cm), except the parameters marked by “eBOD” which were read from a soil database (eBOD, 2013); “DOC” = dissolved organic carbon, “A-horizon max”. = maximum depth of the A-horizon, “water supply” (1 = very low to 7 = very high), “aspect” = deviation from facing south (180 degrees), “usual manure amount / year” (0 = no to 3 = much).

**Table 4:** **Correlation matrix between grub density (all species),** ***Phyllopertha horticola*** **density and environmental and management variables**

	**Grub density**	***P. horticola***	**Humus**	**N**_**min**_	**DOC**	**pH-H**_**2**_ **O**	**Clay**	**Silt**	**Sand**	**A-horizon**	**Humus eBOD**	**Water supply**	**Altitude**	**Slope**	**Aspect**	**Cuts**	**Grazings**	**Manure amount**
Grub density	1																	
*P. horticola*	1.00 **	1																
Humus	0.46*	0.46*	1															
N_min_	0.36	0.36	0.55*	1														
DOC	0.29	0.30	0.49*	0.13	1													
pH-H_2_O	−0.17	−0.15	0.40 t	0.23	−0.11	1												
Clay	−0.14	−0.13	0.27	−0.23	0.14	0.16	1											
Silt	−0.31	−0.30	0.25	0.04	−0.17	0.27	0.50*	1										
Sand	0.27	0.26	−0.29	0.09	0.04	−0.25	−0.83**	−0.90**	1									
A-horizon	0.37	0.37	−0.23	−0.09	−0.45*	−0.07	−0.50*	−0.27	0.43	1								
Humus eBOD	0.37	0.36	0.45*	0.49*	0.07	0.16	−0.02	0.42	−0.26	0.10	1							
Water supply	−0.04	−0.05	0.13	−0.13	0.01	−0.20	0.13	0.11	−0.12	−0.21	0.29	1						
Altitude	−0.06	−0.05	0.14	−0.14	0.01	0.48*	0.24	0.01	−0.11	0.07	−0.01	−0.17	1					
Slope	0.08	0.06	0.17	0.10	0.22	0.20	0.25	−0.00	−0.12	−0.20	0.06	0.10	0.22	1				
Aspect	−0.09	−0.09	−0.31	0.02	−0.06	−0.59*	0.90	0.06	−0.09	0.05	−0.15	0.01	−0.26	−0.17	1			
Cuts	−0.06	−0.08	0.03	0.02	−0.36	0.13	−0.06	0.05	−0.01	0.07	0.15	0.16	0.07	−0.31	−0.25	1		
Grazings	0.09	0.09	−0.06	−0.31	0.50 *	−0.34	0.12	0.10	−0.09	−0.05	−0.10	0.07	0.01	0.47*	0.14	−0.56*	1	
Manure amount	−0.22	−0.23	0.03	0.13	−0.37	0.53*	−0.01	−0.05	0.03	0.08	0.02	−0.24	0.26	−0.37	−0.55*	0.51*	−0.61**	1

The correlation coefficients were calculated across all sites according to Pearson or Spearman in case of the variables “water supply”, “cuts” = usual number of cuts per year, “grazings” = usual number of grazings per year and usual “manure amount” per year. Significant coefficients are marked: * = 0.05 ≥ p > 0.01, ** = p ≤ 0.01. For the definition of parameters see Table 3.

### Relationship between *P. horticola* grub density, management and environmental variables

There was no correlation between *P. horticola* grub density and management variables (Table 4). From the environmental variables, only humus content was significantly correlated to the larval distribution of the species, showing a positive relationship (r = 0.461, p = 0.041).

**Figure 5:**
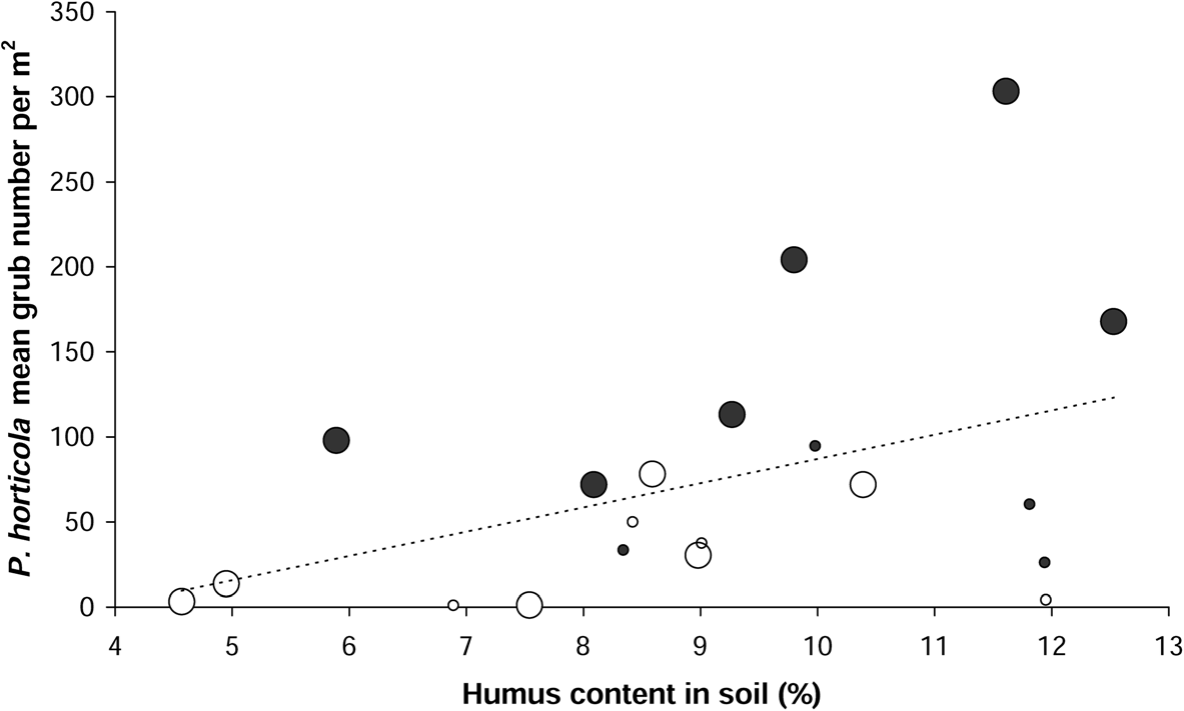
**The relationship between measured humus content, soil depth and** ***P. horticola*** **density.** x-axis: humus content in soil samples (%; 0 – 10 cm soil depth), y-axis: *P. horticola* mean grub number per m^2^, size of circles: maximum depth of A-horizon (small = 15 – 20 cm, large = 25 – 30 cm); R^2^ corr. = 0.38, p = 0.006 according to a multiple linear regression; dashed line = linear regression of *P. horticola* grub density on humus content in the soil; color of circles: white = grub damage had never been observed by the farmers (undamaged sites), dark = sites had recently been affected by grub damage (damaged sites).

The stepwise multiple linear regression analysis resulted in a model, consisting of two environmental variables, explaining 38% of the *P. horticola* density variance (Figure 5). The higher the humus content (in 0 – 10 cm soil depth) and the deeper the A-horizon, the more *P. horticola* larvae were found. As shown in Figure 5, the sites deviating positively from the dashed line (= linear regression between humus content and *P. horticola* density) had a deeper A-horizon (= larger circles) than the sites deviating negatively.

**Figure 6:**
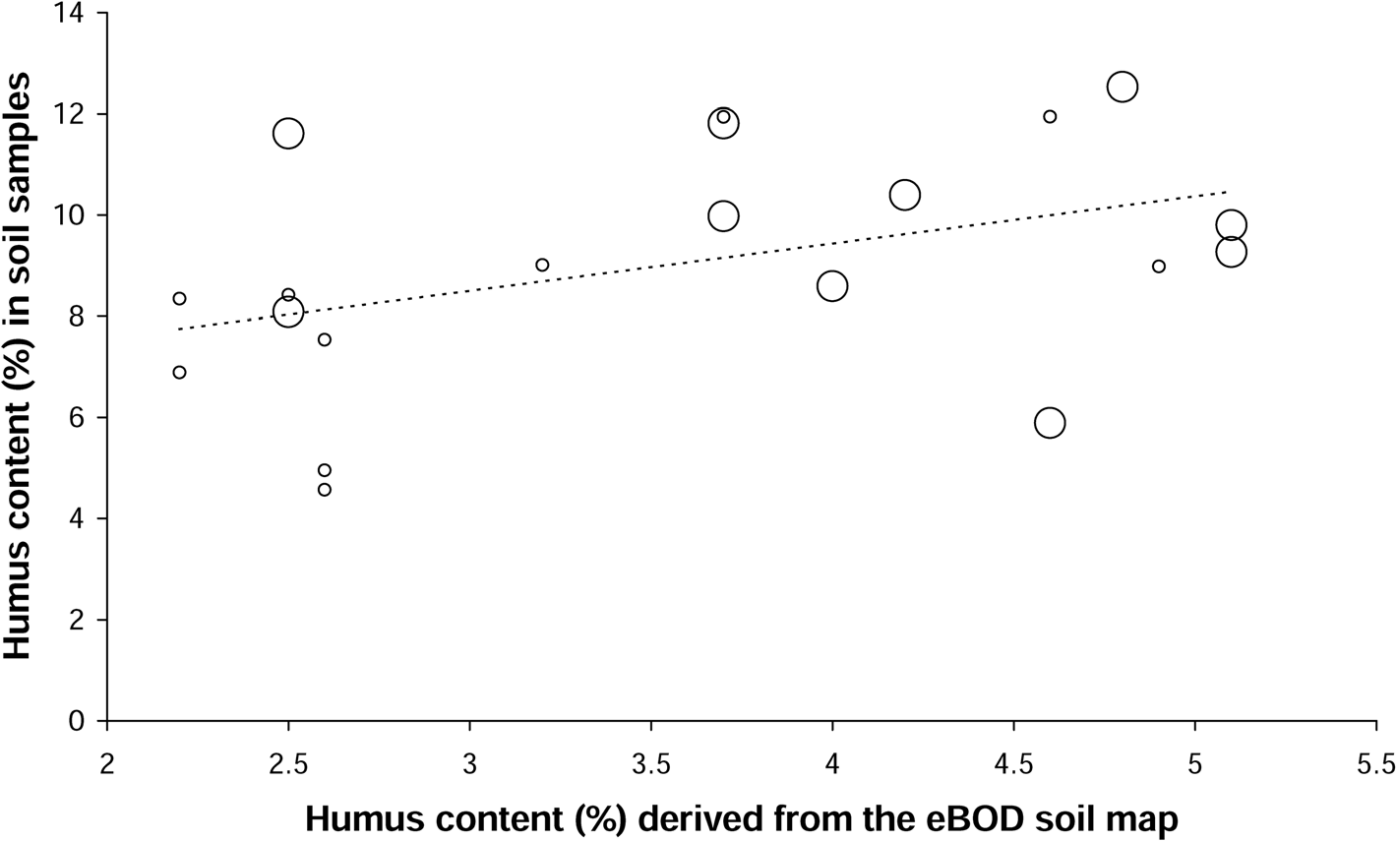
**The effect of grub feeding on the measured humus contents.** Scatter plot showing the relationship between the humus content, derived from the eBOD soil map, and the humus content in the soil samples; small circles: sites with grub densities ≤ 50 individuals/m^2^ (all species), large circles: sites with grub densities > 50 individuals/m^2^; dashed line = regression line for the two humus variables, R^2^ corr. = 0.16, p = 0.046.

The humus contents in the soil samples were constantly higher than the humus contents, derived from the eBOD soil map for the respective site (eBOD, 2013; mean difference = 5.46 ± 2.1%). However, both variables showed a weakly positive, significant relationship (Figure 6). Grub density (= number of grubs per m^2^, all species) provided no additional information for explaining the humus content in the soil samples (p for grubs/m^2^ = 0.140, when added to the regression model). As shown in Figure 6, the small circles (= sites with grub densities ≤ 50 grubs/m^2^) and the large circles (= grub densities > 50 grubs/m^2^) scattered comparably around the dashed linear regression line. Similarly to the humus contents in the soil samples, the eBOD humus contents weakly tended to a positive correlation with grub density (r = 0.366, p = 0.112).

### Probability of grub damage in response to environmental and management conditions

**Figure 7:**
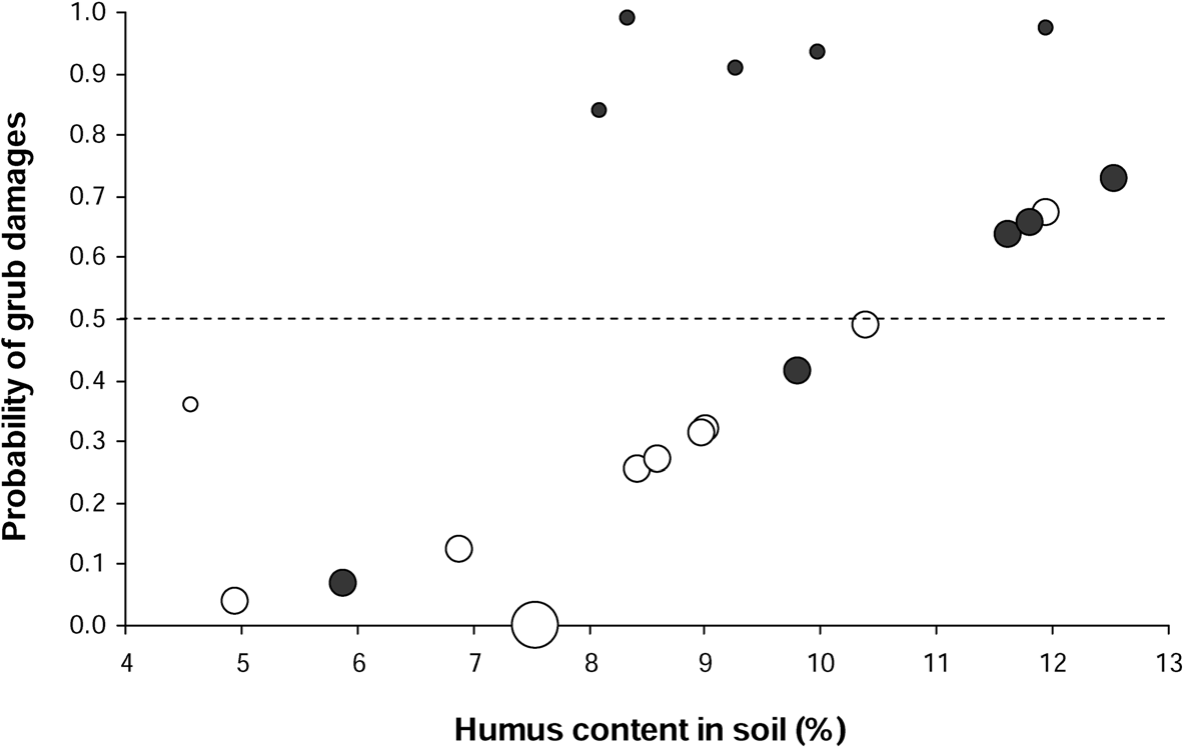
**The relationship between humus content, cutting frequency and grub damage.** x-axis: humus content in soil samples (%, 0 – 10 cm soil depth), y-axis: the probability of grub damage calculated by a multiple logistic regression analysis, size of circles: the usual number of cuts per year (small = 0 – 1 cut, medium = 2, large = 4); color of circles: white = grub damage had never been observed by the farmers – low risk (undamaged sites), dark = sites had recently been affected by grub damage – high risk (damaged site); dashed line = 50% probability that a site belongs to the high risk category.

According to the stepwise multiple logistic regression analysis, the probability that a site belonged to the category that had been recently damaged by grubs (= damaged sites = high risk) was dependent from the humus content in the soil (0 – 10 cm) and the number of cuts per year (Figure 7). The higher the humus content in soil and the lower the usual cutting frequency, the higher the probability of damage was. On basis of this model, 85% of the sites could be correctly classified as recently damaged (= high risk) and undamaged (= low risk) sites, when defining 50% probability as decision limit. As shown in Figure 7, only two recently damaged sites (= dark grey circles) had less than 50% probability (dashed line) and only one undamaged site (= white circles) had more than 50% probability to belong to the high risk category.

## Discussion

Our study provides systematically collected information on grub densities and the dominant grub species at damaged and undamaged mountainous grassland sites in eastern Austria. Additionally, the results contribute to understanding the site and management characteristics that favor high grub densities and the resulting grub damage.

Comparing grub damage histories recorded at the investigated sites with corresponding weather data indicated that grub damage is promoted by drought conditions in the growing season (May to September), resulting from high mean temperatures and comparatively low precipitation sums. This outcome supports several reports in the literature. Studies by Laughlin (1957a, 1964) have shown that high annual mean temperatures promote the development of *P. horticola* larvae and improve the survival prospects of the pupae. Apart from temperature, also proper soil moisture has shown to be important for *P. horticola* grubs (Milne 1964), though exact limits have not been specified yet. Grünbacher et al. (2007) already found that from 2000 to 2006, the heaviest grub damage occurred in the year of heat and drought 2003. By studying meteorological data, it became obvious that the damaged areas in this year were mainly situated in regions with a strong precipitation deficit from January to August 2003 compared to average precipitation sums for this period (Hann et al. 2008). Drought can additionally intensify the effects of grub feeding to the sward by accelerating its withering and delaying its regeneration (Grünbacher et al. 2007). Rising temperatures and intensified drought in the course of climate change as shown in Figure 2 might be responsible for increasingly common grub damages over the past decades in region 1, as reported by the farmers.

*P. horticola* was the most frequent species at the sampling sites (99% of all collected grubs). The rather shallow sampling depth of 10 cm allowed more samples per site, but could have biased the outcome to some degree. First, other grub pest species than *P. horticola* might tend to deeper soil layers, especially when the top soil is occupied by *P. horticola* larvae. Second, the third larval instar of *M. melolontha* starts pupating in deeper soil layers approximately at the end of June (Pötsch et al. 1997, Albert and Fröschle 2010, Kahrer et al. 2011). These grubs would have been missed in the grub survey from September to early October. However, no main flight year of *M. melolontha* was to be expected for the following year 2012 in the investigated regions (Faber 1961, Kahrer et al. 2011). Accordingly, the proportion of third instar larvae in 2011 would have been rather low. Third, under drought stress grubs might migrate into deeper soil regions with more favorable conditions (Ritterhaus 1927, quoted after Milne 1956, Benker and Leuprecht 2005, Albert and Fröschle 2010). Consequently, the densities of some grub species might be underrepresented in our results. But, if sites would have harbored significant populations of other species than *P. horticola*, it would have been quite unlikely that all individuals were located strictly below 10 cm soil depth. According to Keller et al. (2008) grub damage is usually caused by a single species. Therefore, we conclude that *P. horticola* was the dominant species at all sampling sites. This supports observations reported in the literature that this species is largely responsible for damage in Austrian mountainous grassland, while *Melolontha* spp. tend to lower altitudes (Pötsch et al. 1997, Traugott 2003, Benker and Leuprecht 2005).

The grub density threshold, above which damage was visible in 2011, lay at 94 grubs/m^2^. This result corresponds quite well to the threshold value of 100 *P. horticola* larvae/m^2^ recommended for greenkeepers by Bocksch (2003) and Fischer (2007). The highest *P. horticola* grub density in our study was 303 individuals/m^2^. Also Juen and Traugott (2007) described that *P. horticola* can reach densities > 200 individuals/m^2^. Faber (1951b) reported densities even up to 700 individuals/m^2^ during heavy outbreaks. Because of its dominance in our data, regression analyses with environmental and management variables were only conducted for this species. Only at one site in region 1 (Figure 4: farm 9, Table 2), a significant proportion of the collected grubs was determined as *Hoplia philanthus*. High densities of this species were previously detected in Tyrolean cultivated grassland (Traugott and Juen 2008). The fact that this site was the only site with a higher density than 94 grubs/m^2^ in total, but no visible damage in 2011, indicates that *H. philanthus* might be less dangerous for agricultural grassland than *P. horticola*, although Keller and Zimmermann (2005) reported an increasing importance of *Hoplia* spp. in Germany. Ansari *et al.* (2006) considered the species to be a severe pest in Belgian turf.

In 2011, grub damage was only recorded at sites which had also been damaged in preceding years (= damaged sites, high risk). Whereas, the sites at which the farmers had never observed any grub damage (= undamaged sites, low risk), were not damaged in 2011 as well. This consistency confirmed the farmers’ observations on high risk and low risk sites. However, not all high risk sites had grub populations that were strong enough to cause visible feeding damage in 2011. This might reflect the irregular year to year fluctuations of *P. horticola* populations, as shown by Milne (1984). The author described weather conditions, but also enemies (predators, diseases) and intraspecific competition as main factors, responsible for these fluctuations. Even though the population densities are fluctuating, the recently damaged sites had significantly higher grub densities than the undamaged sites, indicating that sites which were classified as high risk sites by the farmers are more suitable habitats for *P. horticola* grub development than low risk sites. Interestingly, at three farms (1, 5, 9) the undamaged sites also revealed significant grub populations. At these sites, the differences to the corresponding damaged sites were quite weak. Obviously, low risk sites, which never show visible grub damage can harbor high *P. horticola* populations. This fact should be considered, when determining where to conduct grub control measures, e.g. the application of entomopathogenic fungi or nematode products (Pernfuss et al. 2005, Strasser 2010).

Since the low and high risk areas of a farm can be situated quite close, local site specific factors must play an important role for the development of high grub densities and the resulting grub damage risk. High risk sites provide adequate conditions for *P. horticola* to maintain relatively high population densities during unfavorable periods, and to produce outbreaks when the fluctuating factors are in the optimum.

### Which site factors are favoring high *P. horticola* grub densities?

Here, the humus content (= organic carbon) in soil is significantly related to *P. horticola* grub density. In soils with higher humus contents, more grubs/m^2^ were found than in soils with lower humus contents. At least, this outcome is valid for the sandy and mostly shallow grounded and sloping sampling sites in this study. The positive correlation corresponds very well to the results of Laznik and Trdan (2014), who also reported a significant relationship between grubs/m^2^ and the content of organic matter. In a former study the same authors already measured a high organic matter content (12.4%) in a Slovenian grassland soil with critical grub densities (Laznik et al. 2012). Raw (1951) found a higher percentage of organic carbon in soils at sites damaged by *P. horticola* larvae than in soils at undamaged sites. The author interpreted the increased organic carbon content at damaged sites as an effect of the grub feeding activity on grass roots. In order to examine this relationship, we compared the humus content in the soil samples with the eBOD humus contents. The latter were derived from a soil map and were therefore unaffected by grub feeding. If grub feeding activity would have been the cause for the increased humus contents at sites with larger grub populations in 2011, the deviations of the soil sample humus contents from the eBOD humus contents would have been connected to the measured grub densities. As shown in Figure 6, the hypothesis of Raw was not supported by our data. On the contrary, the two humus contents even showed a weak positive, but significant correlation and grub density contained no significant information for explaining the residues in this relationship. The deviations between the soil sample and the eBOD humus contents can be attributed to inaccuracies of the soil map, which might not reflect the impact of grassland management on humus content and/or small scale heterogeneity. Similarly to the humus percentages in the soil samples, the eBOD humus values weakly tended to a positive correlation with grub densities, as well. Hence, high humus contents might actually favor the oviposition of *P. horticola* females and/or the development of the larvae. Faber (1951b) observed that *M. melolontha* prefers soils with high humus contents for larval development.

High humus contents in soil can have various beneficial effects on *P. horticola* populations. Milne (1956) concluded from the grassy natural habitat of the species that its main food is grass roots. But McQuillan and Webb (1994) found that the larvae of *Adoryphorus couloni* (Scarabaeidae), which cause increasing damage in southeastern Australian pastures, can selectively feed on concentrated sources of soil organic matter. Kahrer et al. (2011) state, however without references, that young *M. melolontha* larvae feed on fine roots but also on humus particles, which may be valid for young *P. horticola* larvae as well. According to Li and Brune (2005, 2007) humivorous Scarabaeidae larvae can utilize the microbial biomass as well as the nitrogenous components of humus.

Apart from the possible function as food resource for young larvae of *P. horticola*, high humus contents might provide optimal moisture conditions by contributing to the water permeability in soils with higher clay or silt content, but retaining enough soil water in sandy soils (Scheffer et al. 2002), which were typical for the investigated grassland sites. On the one hand, Laughlin (1957b) showed that the eggs are not resistant to desiccation with relative humidities ≤ 98% being lethal. On the other hand, moist soils are inadequate for *P. horticola* grub development (Milne 1964), because of increased pressure by diseases (Milne 1984), difficult hatching conditions (Raw 1951) and the negative effect on soil temperatures. Milne (1964) even considered that:” Apart from proper food, which is provided by grassland, the most important living condition for the garden chafer in the soil appears to be proper soil moisture”. Gaylor and Frankie (1979) showed that *Phyllophaga crinita* (Scarabaeidae), causing heavy damage to crops and turfs in North- and Central-America, did not oviposit in very wet or very dry soil and egg as well as early larval instar survival were low under extreme conditions. Comparably, *Cyclocephala immaculata* (Scarabaeidae), a pest in north-american turf grasses, failed to oviposit and introduced 1^st^ instar larvae did not survive in desiccated turf (Potter and Gordon 1984).

Additionally, due to the darker color humus rich soils are more susceptible to warming by sun radiation (Scheffer et al. 2002). As grubs are poikilothermic, all soil characteristics affecting soil temperature are potentially important factors for grub development. Scheerpelz (1950) supposed that the temperature radiation of soils might even direct swarming females of *M. melolontha* to adequate sites for oviposition. High humus contents might also promote optimal soil structures for the mobility of the larvae and the adults, which deposit their eggs up to 20 cm below the soil surface (Milne 1956).

The second factor, significantly connected to grub density was the maximum depth of the A-horizon. The deeper the A-horizon, the more grubs per square meter could be found. Together, humus content and depth of A-horizon explained 38% of the grub density variation with the highest grub densities in deep humus rich soils. Raw (1951) considered soil depth to affect grub mortality most likely during hibernation in severe winters, but the hypothesis was not supported by his data. Milne (1956) stated that *P. horticola* larvae do “not necessarily hibernate at a deeper level in fields with deeper soil”. Apart from the unclear effect on hibernation depth, deeper soils might simply provide a larger habitat with a more productive root sphere.

### Which management and environmental variables characterize high risk and low risk sites?

Sites that had recently shown grub damage (= high risk) had higher grub densities than sites where grub damage had never been observed by the farmers (= low risk). Accordingly, high risk sites can be considered as better habitats for *P. horticola* development and/or as more attractive locations for oviposition than low risk sites.

When calculated across all sampling sites, the high risk sites had significantly higher DOC (Dissolved Organic Carbon) contents and also tended to higher humus contents than the low risk sites. The humus content was directly positively correlated to grub density, which is corresponding to the observation that high risk areas are better grub habitats. The DOC differences might partly be associated with the humus contents. They might also reflect the larger grub populations at the high risk sites, i.e. their feeding activity, as supposed by Raw (1951). However, DOC contents were not significantly related to the grub densities measured in 2011. Furthermore, neither of the two variables (humus, DOC) was correlated with the estimated amounts of applied manure, queried from the farmers.

Apart from the soil characteristics, the cutting frequency, i.e. the usual number of cuts per year, was significantly lower at the high risk than at the low risk sites. A higher cutting frequency is usually associated with higher amounts of applied manure, as also supported by our data (r = 0.505, p = 0.023). Both management measures promote dense swards which might be unattractive for oviposition and might be more resilient to grub feeding than weak swards (Pötsch et al. 1997, Bocksch 2003). Additionally, a dense sward hinders the warming of the soil by sun radiation and therefore reduces soil temperature. The lack of a significant difference between damaged and undamaged sites concerning the manuring regime might be an effect of inaccurate data on the amount of applied manure per sampling site.

A stepwise logistic regression analysis resulted in a model that uses cutting frequency together with the humus content for predicting grub damage risk. The higher the humus content and the lower the cutting frequency, the higher the probability was that a site belonged to the high risk category. As discussed above, high humus contents might favor the development of large grub populations, while higher cutting frequencies promote dense swards that might reduce oviposition and are more resilient to grub feeding.

Even though we considered various environmental and management parameters, the significant relationships with grub density were few and the correlations were weak. This suggests further unknown factors covering the relationship between grub density and the measured or recorded parameters, e.g. irregular fluctuations of grub populations due to predators, diseases or intraspecific competition (Milne 1984, Juen and Traugott 2007, Laznik et al. 2012). Humus content and cutting frequency might both affect grub density via their influence on soil temperature, a factor which is probably highly important for the soil-dwelling grubs (Laughlin 1964, 1957a, Milne 1984). But soil temperature strongly depends from local weather, which might as well confound the relationship between actual grub densities and site characteristics.

In consideration of these results, we recommend future investigations on the relationship between soil temperature, humus content, depth of A-horizon, cutting regime, grub density and grub damage risk. The actual, small scale soil temperatures and moistures at the sampling sites should be measured with soil sensors and samplings should be conducted over several years to cover grub population fluctuations. Provided that these relationships are well understood, soil temperature models, like CLIMSOIL (Murer et al. 2011, Schaumberger et al. 2013), might enable weather dependent prognoses of grub damage risks in agricultural grassland. Furthermore, we suggest to expand the investigation to the alpine regions in Upper Austria, Salzburg and Tyrol to cover all areas within Austria where damage by grubs occur frequently or regularly.

## Conclusions

The results of this study indicate that *P. horticola* was the dominant species in the top soil layer of the sampled sites and was largely responsible for grub damage in the investigated region, which was situated at 800 – 1,200 m above sea level and characterized by lime-free, sandy Leptosols or Cambisols over siliceous, gravelly material. The damage threshold for *P. horticola* lay at 94 grubs/m^2^. Also sites which had not shown grub damage revealed significant grub populations, which should be considered when applying control measures. Regression analyses indicated high humus contents in soil to be favorable for the development of high grub densities in the sampling sites, while intensive cutting frequencies might have mitigating effects on grub damage.
